# The Mastitis Pathogens Culture Collection

**DOI:** 10.1128/MRA.00133-19

**Published:** 2019-04-11

**Authors:** Simon Dufour, Josée Labrie, Mario Jacques

**Affiliations:** a Mastitis Network, Faculté de Médecine Vétérinaire, Université de Montréal, Saint-Hyacinthe, Quebec, Canada; University of Maryland School of Medicine

## Abstract

The Mastitis Pathogen Culture Collection contains more than 16,000 mastitis-causing bacterial isolates from milk samples taken from cow quarters and bulk tanks in a national cohort of 91 dairy farms across Canada over a period of 2 years. These isolates are linked to demographic and production data that were recorded at the mammary gland, cow, and farm levels.

## ANNOUNCEMENT

Mastitis is an inflammation of the udder that affects a high proportion of dairy cows throughout the world (1). Mastitis differs from most other animal diseases in that several diverse kinds of bacteria are capable of infecting the udder. These pathogens invade the udder, multiply, and produce harmful substances that result in inflammation, reduced milk production, and altered milk quality. Mastitis imposes considerable and recurring economic losses on the dairy industry worldwide (2).

Microorganisms that most frequently cause mastitis can be divided into two broad categories, as follows: contagious pathogens, which are spread from cow to cow, primarily during the milking process, and environmental pathogens, which are found throughout the habitat of dairy cows (1). The predominant contagious pathogens are Staphylococcus aureus, Streptococcus agalactiae, and Corynebacterium bovis, while the predominant environmental pathogens are Escherichia coli, Streptococcus uberis, Streptococcus dysgalactiae, and other Gram-positive and catalase-negative cocci (here, “other streptococci”). Staphylococci other than Staphylococcus aureus, often described in the past as coagulase-negative staphylococci, are also important mammary gland pathogens. The epidemiology of the latter pathogens, however, is still under investigation and varies by staphylococcal species, with some species possibly being more opportunistic pathogens, while others are likely to be host-adapted pathogens.

The Mastitis Pathogen Culture Collection (MPCC) provides access to bacteria isolated from milk samples taken from cow quarters and bulk tanks in a national cohort of 91 dairy farms across Canada over a period of 2 years in 2007 and 2008. This cohort study was conducted by the Mastitis Network (formerly known as the Canadian Bovine Mastitis and Milk Quality Research Network). A complete description of the herd selection process as well as of the characteristics of these herds has been published previously (3). During that study, mammary gland quarters from recruited cows were sampled repeatedly, with some glands being sampled more than 20 times over the 2-year period. This sampling scheme allowed for monitoring the persistence of intramammary infections and for a comparison of isolates recovered from a same mammary gland, cow, or herd over time. Demographic and production data were recorded at individual cow and farm levels. Health management data are documented, and extensive questionnaire data detailing farm management and cleanliness information are also captured. Just fewer than 133,000 milk samples were collected, and bacterial cultures were performed. The MPCC archives the isolates recovered from intramammary infections of cows in the cohort and currently holds more than 16,000 bacterial isolates (Table 1) which are accessible to researchers.

**TABLE 1:** Bacterial isolates of the Mastitis Pathogen Culture Collection^*a*^

Bacterial species	No. of isolates
*Corynebacterium* spp.	1,349
Escherichia coli	496
*Enterobacter* spp.	58
*Klebsiella* spp.	194
*Nocardia*	14
Other Gram-negative species	125
Other Gram-positive species	105
Staphylococcus aureus	4,233
Staphylococcus hyicus	120
*Staphylococcus* spp. (coagulase negative)	6,929
Streptococcus uberis	561
Streptococcus agalactiae	1
Streptococcus canis	3
Streptococcus dysgalactiae	514
*Streptococcus* spp.	1,499
Trueperella pyogenes	137
Total	16,338

a The collection also contains fungi (*n* = 6), *Prototheca* spp. (*n* = 29), and yeast (*n* = 127).

One interesting and unique feature of this collection of bacterial strains is that each isolate is linked to the data regarding its cow host (type of infection, duration, clinical signs, treatments, parity, etc.), the on-farm management practices, and, for 200 cows, the host’s DNA.

### Data availability.

Freeze-dried isolates and data on the quarters (e.g., position, teat score, and somatic cell count), cows (e.g., parity, breed, and milk production), and herds (housing type, demography, and management) are available for Canadian and international scientists. Data describing characteristics of many of the isolates (e.g., antimicrobial resistance profile, gene sequencing, whole-genome sequencing, and *spa* type) are also available. Requests for the resource should be directed to the corresponding author.
